# Supplementary material for: Two Avastrovirus Species Discovered in Psittaciformes Expand the Host Range of the Family Astroviridae
Source: Viruses. 2025 Mar 20;17(3):450. doi: 10.3390/v17030450 (PMC11946394; doi:10.3390/v17030450)
Supplement: Supplementary file 1 [file viruses-17-00450-s001.zip › viruses-3483263-supplementary.pdf]

Supplementary Figure S1

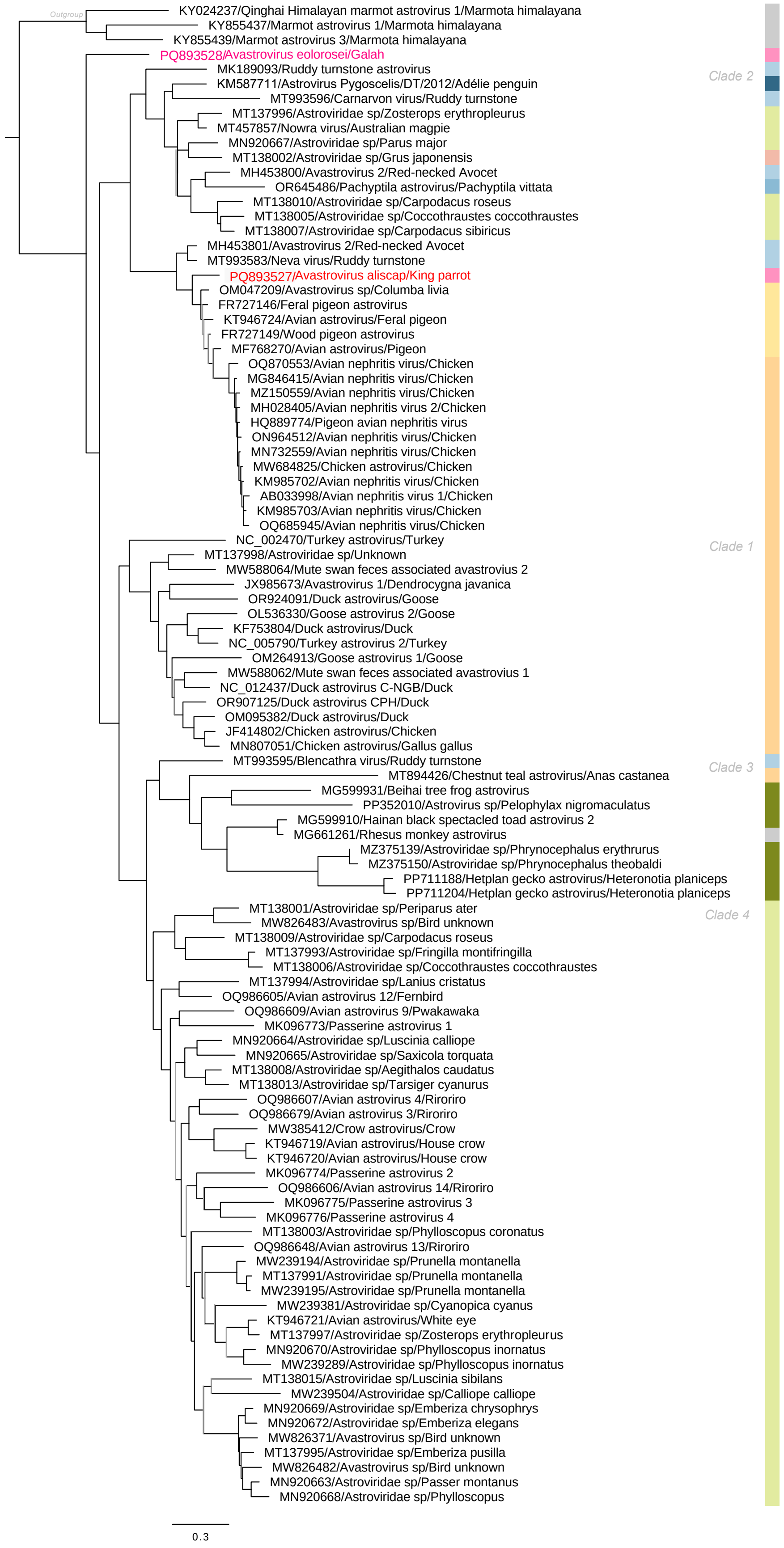

Figure S1. Expanded maximum likelihood phylogeny of the RNA dependent RNA polymerase of avian astroviruses. The tree was generated from an alignment of 250 amino acids of the Astroviridae RdRp domain (cd23172) in IQ-TREE 2 using ModelFinder with 1000 Ultrafast bootstrap supports. The tree is rooted on a marmot astrovirus outgroup. Nodes with fewer than 70% support are drawn in pale grey. GAstV and KPAstV are shown in pink and red text, respectively. Phylogenetic clades that are consistent across both domains are shaded with grey boxes. Host association for each sequence is summarised in panels adjacent to each tree with colours representing avian orders and non-avian classes: Reptilia and Amphibia (dark green), Mammalia (grey), Anseriformes and Galliformes (orange), Charadriiformes (pale blue), Columbiformes (yellow), Gruiformes (pale red), Passeriformes (pale green), Procellariiformes (blue), Psitticiformes (pink) and Sphenisciformes (dark blue).

Figure S2

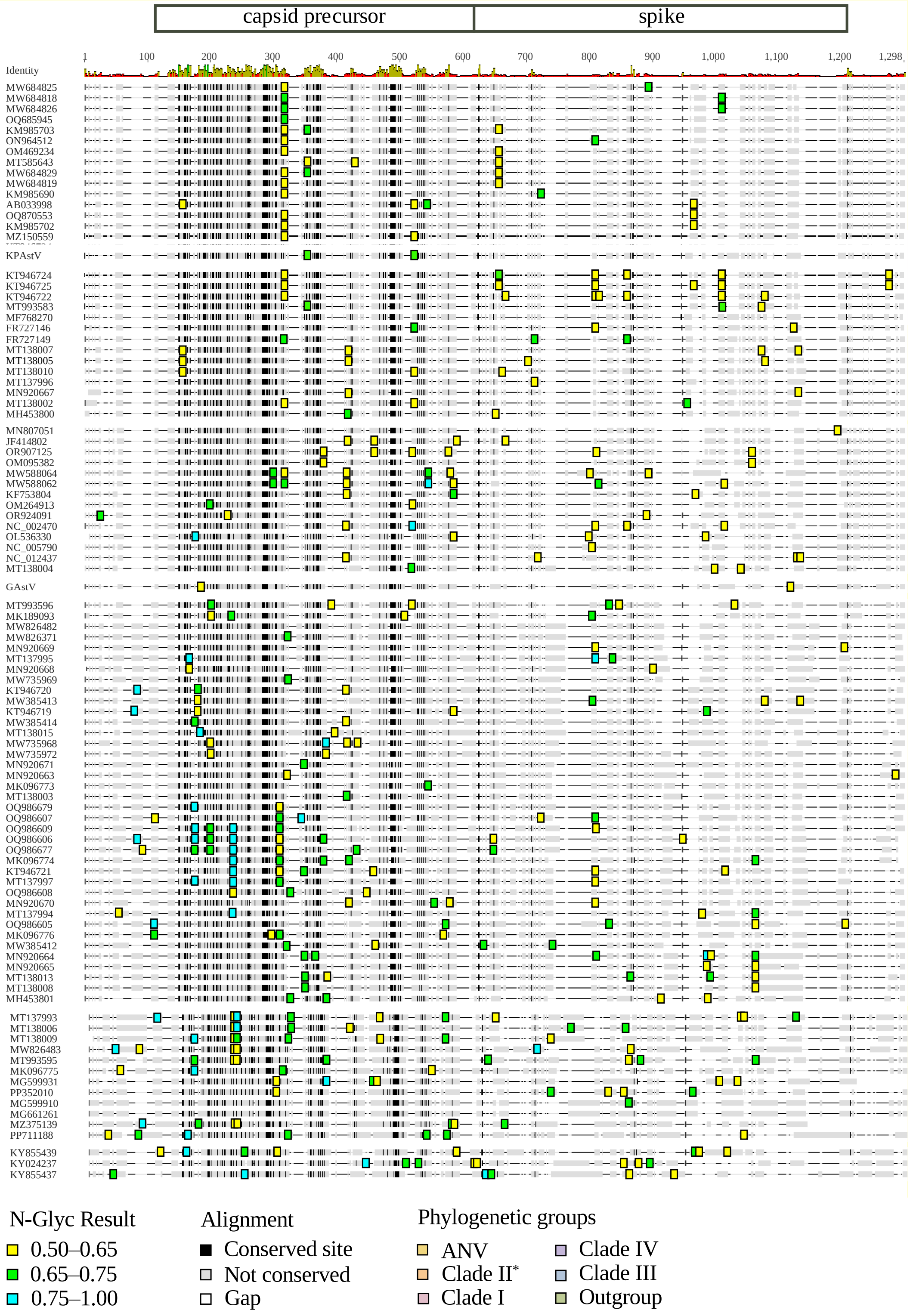

Figure S2. Predicted N-glycosylation in *Avastrovirus* ORF2 translated protein. A complete ORF2 amino acid alignment from *Avastrovirus* representatives was generated with the E-LNS-i algorithm in MAFFT. The position of the two key astrovirus ORF2 protein domains, capsid precursor and spike, of Turkey astrovirus 2 are denoted by a bar above the alignment. N-glycosylation tetrapeptides (N-X-S/L-X) were predicted in NetGlyc-N and results are annotated on the alignment according to level of confidence (width not to scale of alignment residues).

The alignment is vertically truncated according to phylogenetic clustering observed in Figure 2. NCBI GenBank accessions are provided for each sequence and background colours of each cluster correspond to ANV strains (yellow), non-ANV Clade II (orange), Clade I (pink), Clade IV (purple), Clade III (blue), and the Marmot astrovirus outgroup (green). The novel GAstV and KPAstV are isolated from the alignment to aid visual comparison. *KPAstV phylogenetically clusters within Clade II but has been isolated to the aforementioned effect.

Supplementary Table S1. SWISS-MODEL results for *Avastrovirus* spike proteins. Models were built based on a template of the crystal structure of TAstV-2 spike protein (PDB #3ts3.1.A). The table provides Global Model Quality Estimates (GMQE), which , and QMEANDisCo global scores.

Virus	Accession*	Host	Geographic source	GMQE	QMEANDisCo Global	Sequence Identity to TAstV-2 (%)	Similar structure**
Avastrovirus aliscap	PQ893527	King Parrot	Australia	0.22	0.33±0.05	11.19	KPAstV-like
Feral pigeon astrovirus	FR727146	Feral pigeon	Norway	0.38	0.33±0.05	7.09	KPAstV-like
Avian astrovirus	MF768270	Red-necked avocet	Australia	0.36	0.32±0.05	6.29	KPAstV-like
Wood pigeon astrovirus	FR727149	Wood pigeon	Norway	0.35	0.33±0.05	10.64	KPAstV-like
Avastrovirus 2	MH453800	Red-necked avocet	Australia	0.38	0.34±0.05	11.27	KPAstV-like
Avian astrovirus	KT946724	Feral pigeon	Hong Kong	0.46	0.4±0.05	16.22	TAstV-2-like
Neva virus	MT993583	Ruddy turnstone	Australia	0.44	0.42±0.05	14.89	TAstV-2-like
Avian nephritis virus	OQ870553	Chicken	Italy	0.47	0.41±0.05	17.46	TAstV-2-like
Avian nephritis virus	AB033998	Chicken	Japan	0.46	0.41±0.05	19.05	TAstV-2-like
Chicken astrovirus	MW684825	Chicken	Netherlands	0.47	0.39±0.05	13.23	TAstV-2-like
Turkey astrovirus 1	NC_002470	Turkey	United States	0.44	0.46±0.05	20.63	TAstV-2-like
Duck astrovirus CPH	OR907125	Duck	China	0.38	0.36±0.05	23.9	TAstV-2-like
Mann-Whitney U test between KPAstV-like and TAstV-2-like structures				*p<0.001*	*p<0.01*	*p<0.01*	

*No templates found for the following accessions: PQ893528 (GAstV), MK096773, MT993595, PP711188;

**See Figure 3
